# Comprehensive analysis of supervised learning methods for electrical source imaging

**DOI:** 10.3389/fnins.2024.1444935

**Published:** 2024-11-27

**Authors:** Sarah Reynaud, Adrien Merlini, Douraied Ben Salem, François Rousseau

**Affiliations:** ^1^IMT Atlantique, LaTIM U1101 INSERM, Brest, France; ^2^IMT Atlantique, Lab-STICC UMR CNRS 6285, Brest, France; ^3^Univ Brest, CHU Brest, LaTIM UMR 1101, Brest, France

**Keywords:** electroencephalography, deep learning, inverse problem, neuroimaging, data simulation

## Abstract

Electroencephalography source imaging (ESI) is an ill-posed inverse problem: an additional constraint is needed to find a unique solution. The choice of this constraint, or prior, remains a challenge for most ESI methods. This work explores the application of supervised learning methods for spatio-temporal ESI, where the relationship between measurements and sources is learned directly from the data. Three neural networks were trained on synthetic data and compared with non-learning based methods. Two distinct types of simulation, each based on different models of brain electrical activity, were employed to quantitatively assess the generalization capabilities of the neural networks and the impact of training data on their performances, using five complementary metrics. The results demonstrate that, with appropriately designed simulations, neural networks can be competitive with non-learning-based approaches, even when applied to previously unseen data.

## 1 Introduction

Electroencephalography (EEG) is a very popular method to characterize brain activity by recording differences in electric potential on the scalp using electrodes. Its popularity arises from the fact that it provides non-invasive, millisecond-resolved information that can be related to the electrical activity in the brain. The electrical sources that generate the scalp potential distribution are macrocolumns of similarly active neurons, typically modeled by current dipoles (Nunez and Srinivasan, 2006). Numerical models of the relationship between a current distribution modeling the brain activity and the potential values at the electrode's positions can be obtained by solving the EEG forward problem multiple times and forming a leadfield matrix (Hallez et al., 2007). EEG source imaging (ESI) is an inverse problem that aims to estimate the unknown brain activity (or current source distribution) from known EEG measurements. Accurately solving the EEG inverse problem would provide a recording of the brain activity that would be highly resolved in time and space, something that very few, if any, neuroimaging modalities can achieve simultaneously. The EEG inverse is however ill-posed, due to, among other things, the significantly larger number of sources in the brain than electrodes and the issues of both superposition and volume conduction. This ill-posedness causes several solutions of the problem to be admissible making it challenging to select the one that corresponds to the actual brain activity. To render the problem uniquely solvable, it is necessary to provide prior information on the data to be estimated.

Two main families of methods exist to solve the ESI problem: parametric and non-parametric methods (Grech et al., 2008; Baillet et al., 2001). In parametric methods, only a few dipoles are assumed to be active, resulting in fewer unknowns to estimate, but making a strong assumption on the source distribution. Non-parametric or distributed source methods, on the other hand, aim at estimating the activity of numerous dipoles placed on a predetermined grid. Variational formulations, which incorporate a regularization term as prior on the data to be estimated, fall into this last category. Although various types of regularization have been proposed in literature (Hämäläinen and Ilmoniemi, 1994; Uutela et al., 1999; Pascual-Marqui et al., 2011), estimating some source activities remains challenging. This includes current distributions corresponding to extended sources with EEG topography similar to that of a single dipole, or multiple sources active at the same time. Moreover, these methods require manual setting of strong priors on data, which is challenging at best.

In this context, the use of learning based methods, more specifically neural networks (NNs), offers a way to learn the inverse function directly from the data. Among these, supervised learning is a promising strategy, but requires ground truth data. In the absence of a comprehensive dataset encompassing whole-brain activity and corresponding EEG recordings, synthetic, simulated, data serves as a valuable substitute, provided that the simulation strategy accurately represents the complexities of real EEG data to ensure the network's ability to generalize to actual subject recordings. A common challenge in employing learning-based approaches lies in identifying an appropriate data representation and neural network architecture that effectively capture the underlying patterns in the data. NN-based approaches have been used to estimate the spatial distribution of sources at a specific time point, using multi-layer perceptron (MLP) or two-dimensional convolutional NN (2D-CNN) (Wei et al., 2021; Pantazis and Adler, 2021; Hecker et al., 2021). In this work, we focus on estimating the spatial distribution of brain activity not only at a single moment, but over a sequence of time points, which we refer to as “spatio-temporal ESI.” To effectively capture the spatio-temporal dynamics of the data, previous studies have employed architectures such as LSTMs (Hecker et al., 2022), autoencoders (Liang et al., 2023), transformers (Zheng and Guan, 2023), and spatio-temporal networks composed of LSTMs and fully connected layers (Sun et al., 2022).

The paper is structured as follows: Section 2.1 describes the forward model, inverse problem resolution, and the use of standard non-learning and learning based methods. Section 2.2.1.2 and Section 2.2.1.3 present two different approaches to generate realistic synthetic electrophysiologic data. Additionally, Section 2.2.2 includes details of the real data used to study the generalization of neural networks to real data. Section 2.2.3 details the neural networks' architectures and the training setup used. Section 2.2.4 presents the evaluation metrics used to assess and compare the performance of the different methods on different aspects of the result. Finally, Section 3 presents the outcomes of various experiments, including the estimation of multiple extended sources on two different types of synthetic data (Section 3.1), an initial study on out-of-domain generalization (Section 3.2) to evaluate the performance of a trained network on another synthetic dataset, and lastly, the investigation on real data of visually evoked potential (Section 3.3).

## 2 Methods

### 2.1 EEG source imaging

#### 2.1.1 Forward model

The activity measured by the EEG arises mainly from macrocolumns of pyramidal cells in the cerebral cortex (Baillet et al., 2001). Such macrocolumns, or similarly activated macrocolumns, can be modeled by an equivalent current dipole (ECD) (Nunez and Srinivasan, 2006) defined by its position, orientation, and magnitude. Since there are many such macrocolumns in the brain, the commonly used model for brain activity in ESI is based on the distribution of ECDs.

For an ECD with given position, orientation and unit magnitude, the potential values, at given positions, created by the ECD can be computed by solving Poisson's equation on a model of the subject's brain called a head model (Hallez et al., 2007). By repeating this operation for each ECD of interest in the brain, we can build the leadfield matrix **L**. One coefficient *l*_*i, j*_ of this matrix corresponds to the potential value at electrode *i* created by the *j*^*th*^ dipole.

The estimation of the leadfield matrix requires an accurate head model of the subject and an appropriate solver. The head model consists of a equivalent source space and an electrode montage. The source space is based on the subject's anatomy that can be obtained from a segmented MRI volume (e.g., white matter, gray matter, skull and scalp). From this segmentation, the equivalent dipole sources are placed at various positions within the head, for instance on a sub-sampled mesh of the brain or on a regular grid within the head. The electrode montage corresponds to the number and position of the electrodes and must be co-registered to the source space (Michel and Brunet, 2019).

Analytic solutions to the forward problems only exist for spherical head models. For more realistic head models, numerical solvers based on the Finite Element Method (FEM) and Boundary Element Method (BEM) are typically used (Hallez et al., 2007). The FEM or BEM rely on anatomical meshes and additional biophysical information such as the electric conductivity of the different tissues. Gathering subject specific anatomical and functional information, the leadfield matrix defines the mapping between a brain activity modeled by a distribution of ECDs to the EEG values as


(1) $$\begin{array}{l} \text{Y}=\text{LX}+\epsilon , \end{array}$$

where $\text{X}\in {\mathbb{R}}^{N_{s}\times T}$ is the current distribution, with *N*_*s*_ sources and *T* time points, $\text{Y}\in {\mathbb{R}}^{N_{e}\times T}$ is the potential distribution at *N*_*e*_ electrode positions on the scalp, and $\epsilon \in {\mathbb{R}}^{N_{e}\times T}$ represents some additive noise. See for instance (Michel and Brunet, 2019) for more details regarding head modeling.

#### 2.1.2 Inverse problem

One of the sources of ill-posedness of the EEG inverse problem—required for ESI—is the high number of unknown sources to be imaged in comparison to the low number of—noisy—observations (tens of electrodes). There are two main categories of methods used to solve the inverse problem in EEG: parametric or equivalent current dipole methods and non-parametric or distributed source methods (Grech et al., 2008; Baillet et al., 2001; Maksymenko, 2019).

Parametric methods assume a limited number of active dipoles with unknown parameters to be estimated (the positions, amplitudes and possibly orientations of these dipoles). In this situation, the leadfield matrix **L** is parameterised by the positions and orientations of the dipoles. This leads to a non-linear problem. This category of methods includes dipole fitting (e.g., nonlinear least squares) and dipole scanning [e.g., beamforming (Van Veen et al., 1997), MUSIC (Schmidt, 1986)] approaches.

In this work, we focus on non-parametric methods aimed at estimating the amplitudes of sources placed at fixed locations. The leadfield is pre-computed which leads to a linear problem. This category includes methods based on a variational formulation of the form


(2) $$\begin{array}{l} \hat{\text{X}}=\arg\underset{\text{X}}{\min}\left\{||\text{Y}-\text{LX}|{|}_{F}^{2}+\lambda R\left(\text{X}\right)\right\}, \end{array}$$

where a prior is assumed on the source data in the form of a regularization term *R*(*X*) weighted by a regularization parameter λ. Here, ||.||_*F*_ denotes the Frobenius norm.

This formulation is a trade-off between the data fidelity term $||\text{Y}-\text{LX}|{|}_{F}^{2}$, which ensures that the estimated source distribution matches the observed data, for the given leadfield matrix, and the regularization term, which encodes a prior on the data to be estimated. For example, in the Minimum Norm Estimate (MNE) solution (Hämäläinen and Ilmoniemi, 1994), the regularization term is the *l*^2^ norm of the sources. This regularization function corresponds to the estimation of the solution with minimal energy. This can lead to (over)smooth estimates and poor estimation of deep sources. To address this limitation, other methods have been proposed, such as weighted MNE or LORETA (Pascual-Marqui et al., 2011), which uses the *l*^2^ norm of a weighted source distribution. Another commonly used method is standardized low-resolution brain electromagnetic tomography [sLORETA (Pascual-Marqui et al., 2002)], in which the MNE estimate is rescaled using the noise covariance of the data. *l*^1^ norms can also be used as a prior to enforce spatial sparsity on the solution (Uutela et al., 1999). The same procedure of adding a weight matrix within the *l*^1^ norm can be used (Candes et al., 2008).

Several other regularization functions have been proposed to improve the estimation of different activities. Still, many challenges and limitations remain in the use of such methods. The choice of a prior is not trivial, especially for brain activity, which is very complex and can be significantly different depending on the cognitive state of the subject. These methods also require the setting of hyperparameters, such as the regularization parameter λ in variational formulations. Due to these limitations, the work presented here focuses on learning based methods to estimate the inverse function directly from the data.

#### 2.1.3 Learning based methods

Artificial Neural Networks (ANN) are effective tools for learning arbitrarily complex functions, such as the inverse function between the equivalent source distribution representing the brain activity and the EEG data. Existing works in the literature focus on estimating the inverse function in a supervised framework, in which the ground truth (i.e., brain activity) is known. First of all, we need to consider the type of task that needs to be solved by learning. Early work using neural networks focused on estimating the location of a few active dipoles (Zhang et al., 1998; Yuasa et al., 1998; Van Hoey et al., 2000), similar to the parametric methods presented previously. More recent work aims at estimating the amplitude of sources placed on a predefined grid, similarly to what is done in distributed schemes. In this category, some studies estimate the activity for a single time point, either from a single EEG time point (Razorenova et al., 2020; Wei et al., 2021; Hecker et al., 2021; Guo et al., 2022), or from multiple EEG time points (Sun et al., 2020; Pantazis and Adler, 2021). A more general approach is to consider a spatio-temporal regression approach, to estimate the amplitude of the sources for all time samples from the EEG data measured. In this work, we focus on this task, called spatio-temporal ESI, as such a versatile framework would help to acquire a better understanding of the brain activity whatever the acquisition context may be, by providing accurate spatial and temporal information.

The performance of deep neural network approaches depends on the representation of the data within the architecture. Recent works for spatio-temporal ESI have therefore focused on the design of neural network architectures to exploit both the spatial and temporal dimensions of the data. Transformers (Zheng and Guan, 2023) and LSTMs (Hecker et al., 2022) are good candidates for exploiting the temporal structure of EEG data. Based on the LSTM architecture, Jiao et al. (2022) further investigate smooth spatial constraints on sources using Graph Fourier Transform (GFT). In Huang et al. (2022), propose a denoising autoencoder with separate layers for the spatial and the temporal dimension of the data. In Sun et al. (2022), the authors present a method called deepSIF relying on a two-block network architecture, consisting of a spatial block (resNet with fully connected layers) and a temporal block (multiple LSTM layers).

**Figure 1:**
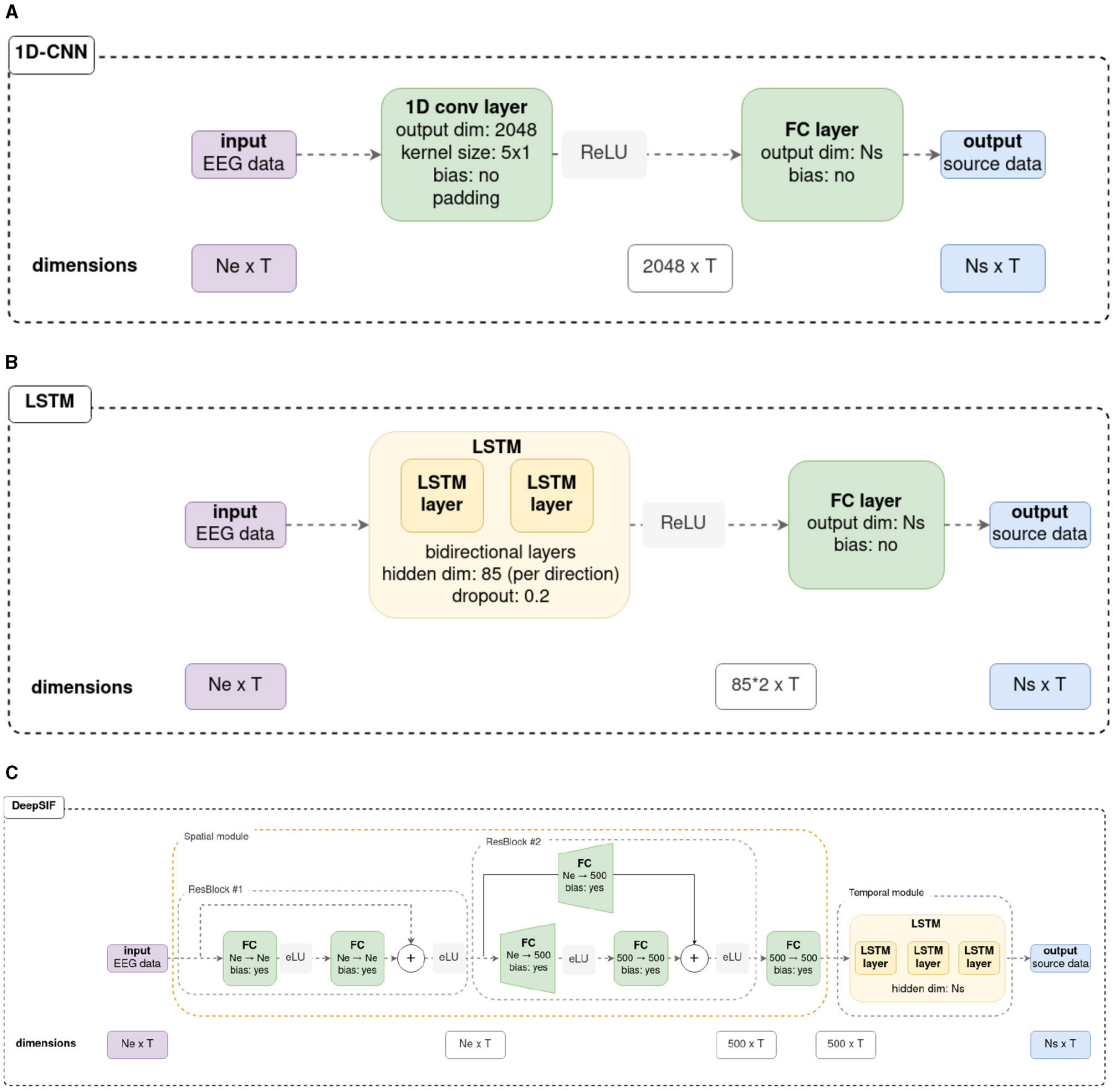
Details of the three neural networks used in this work. Original schemes for the LSTM and deepSIF architectures can be found in there original articles (Hecker et al., 2022; Sun et al., 2022). For the neural networks in Sections 3.1, 3.2, *N*_*e*_ = 90, *N*_*s*_ = 994 and *T* = 500. For the neural networks trained in Section 3.3, *N*_*e*_ = 59, *N*_*s*_ = 1, 176, and T = 500. **(A)** 1D-CNN architecture details. **(B)** LSTM from Hecker et al. (2022) architecture details. **(C)** deepSIF, spatio-temporal network from Sun et al. (2022) architecture details.

In this work, we investigate the use of three architectures for spatio-temporal ESI: one-dimensional wide CNN (1D-CNN), LSTM, and deepSIF—a spatio-temporal network proposed in Sun et al. (2022). Figure 1 details the architectures, which are described in the following. The code used to define and train the neural networks mentioned here is available on our github: https://github.com/SarahReynaud/stESI_pub.

The 1D-CNN architecture makes use of temporal convolutions applied across all the measured signals. The signals acquired from the electrodes correspond to the *channels*. This architecture then exploits all the spatial information within time windows defined by the size of the convolution kernels. This wide network is composed of a convolutional layer of 2,048 filters with a kernel of dimension *N*_*e*_×5 to account for all electrodes in the input data. It is followed by a dense layer that outputs the activity of the sources. A rectified linear unit (ReLU) activation function is used between the convolutional and the dense layer.

The second network studied is based on a LSTM architecture, reproducing the one described in Hecker et al. (2022). It consists of two bi-directionnal LSTM layers and a dense layer that allows the activity of each source of the head model to be estimated from the features learned by the LSTM. As in the original paper, the LSTM layers have a hidden state dimension of 85 (per direction of the LSTM), and dropout is employed in the LSTM with a rate of 0.2. A ReLU function is employed between the LSTM and dense layers.

The third network is the deepSIF network from Sun et al. (2022). It consists of two modules: a spatial module and a temporal module. The spatial module is based on residual blocks. Each residual block is composed of two dense layers, with an exponential linear unit (eLU) activation function, and a skip connection from the input of the block to the output of the second dense layer. The features extracted from the initial residual block are of the same dimension as the input tensor, whereas the second residual block increases the spatial dimension of the data from *N*_*e*_ to 500. As the dimension is modified between the input and the output of the block, a dense layer is employed in the skip connection to augment the dimension of the input data. After the two residual blocks, a dense layer of 500 neurons is used. Then the temporal module comprises three LSTM layers with a hidden state of dimension *N*_*s*_. The output of the LSTM corresponds to the estimated source activity, which has a dimension of *N*_*s*_×*T*. If necessary, the original architectures were adapted to align with the number of input electrodes or output sources (input and output dimensions) corresponding to the used head models. Otherwise, the parameters employed were those originally described in the source articles. Further details can be found in the Figure 1, and in the original articles.

For a fair comparison of these neural networks, the same loss function has been used. We have observed experimentally that using standard loss function like the Mean Squared Error can lead to numerical issues due to the small value of the amplitudes of the source data and to the high number of points in a sample. The loss function used in this study is a cosine similarity loss $L_{cs}$ defined as


(3) $$\begin{array}{l} L_{cs}\left(\text{X},\hat{\text{X}}\right)=-\frac{1}{T}\overset{T}{\underset{k=1}{\sum }}\left(\frac{\sum_{i=1}^{N_{s}}{\text{X}}_{i,k}{\hat{\text{X}}}_{i,k}}{||{\text{X}}_{:,k}||||{\hat{\text{X}}}_{:,k}||}\right). \end{array}$$

Using this cost function allows for a better reconstruction of the pattern of source amplitude at each time point, regardless of the exact estimated amplitude. The cosine similarity loss function needs a scaling process to restore the correct amplitude values. This processing is done via the use of Global Field Power (GFP) scaling (Hecker et al., 2022).


(4) $$\begin{array}{l} {\hat{\text{X}}}_{:,t}={\tilde{\hat{\text{X}}}}_{:,t}\frac{std\left({\text{Y}}_{:,t}\right)}{std\left({\tilde{\hat{\text{Y}}}}_{:,t}\right)}, \end{array}$$

where $\tilde{\hat{\text{Y}}}$ is the estimated EEG data obtained by re-projecting the estimated source distribution on the electrode space using the forward model $\tilde{\hat{\text{Y}}}=\text{L}\tilde{\hat{\text{X}}}$.

EEG data and source data are normalized by calculating $\tilde{\text{Y}}=\frac{\text{Y}}{\alpha }$ and $\tilde{\text{X}}=\frac{\text{X}}{\alpha }$, where α = max(|**Y**|). Such a data normalization preserves the linear relationship between the measurements and the source distribution.

### 2.2 Experimental setup

In the absence of ground truth for spatio-temporal ESI, evaluation relies on simulated data. In this context, the synthetic data must capture the complexities of real EEG data. To prevent evaluation biases introduced by the simulated dataset, we should employ multiple simulation strategies to assess the models' ability to generalize beyond training data. To this end, we propose to use in this work two data simulation methods to achieve a thorough evaluation of inversion methods.

#### 2.2.1 Data simulation

This section describes the head model used to compute the leadfield matrix, and simulations based on physical and biophysical models of brain activity.

##### 2.2.1.1 Head model

The head model is composed of an electrode montage and a source space. We employed the template MRI fsaverage, from FreeSurfer and accessible in the python library mne-python (Gramfort et al., 2013), to construct the source space. To calculate the leadfield matrix, we used BEM-based solver, with three homogeneous tissues: brain, skull, and skin, with conductivity values of 0.3 S m^−2^, 0.006 S m^−2^ and 0.3 S m^−2^. To generate the source space, the brain mesh is subsampled using “icosahedron 5” subsampling, resulting in a mesh of 20,484 vertices.

Following Sun et al. (2022), we use cortical regions defined in The Virtual Brain (TVB) (Sanz Leon et al., 2013) and employed the center of mass of each region as the source location for estimation, leading to a source space of 994 regions. The sources dipolar orientations were fixed perpendicular to the surface.

The “*standard_1020*” electrode montage from the mne-python package is coregistered with fsaverage head and consists of 90 electrodes (originally 94, but 4 were removed due to duplication resulting from Modified Combinatorial Nomenclature (MCN)).

From the initial source space containing 20,484 sources and the electrode montage, a leadfield matrix ${\text{L}}_{\text{S}}\in {\mathbb{R}}^{N_{e}\times 20484}$ is computed. Then to obtain a leadfield corresponding to the regional source space, based on the assumption that all sources in the region have equal activity (Sun et al., 2022), a coefficient for an electrode *i* and a region *R*_*j*_ is obtained by taking the sum of the coefficient for electrode *i* for all the sources *j*′ in the region *R*_*j*_.

As a result, the size of the leadfield matrix **L** is *N*_*e*_×*N*_*s*_ where *N*_*e*_ = *90* and *N*_*s*_ = 994.

To generate pairs of EEG and source activity signals, we first generate a source distribution and then use the leadfield matrix and the addition of noise to obtain the corresponding EEG data. In this work, we investigate two state-of-the-art source generators: SEREEGA and Neural Mass Models (NMM).

##### 2.2.1.2 Simulation based on physical models (SEREEGA)

SEREEGA (Krol et al., 2018) is a Matlab toolbox that uses physical models derived from the observations of different event-related brain activity profiles to simulate source distributions according to a spatial pattern (number and locations of active sources) and a temporal pattern (waveform of each of the sources). In this work, we simulate extended regions of active sources consisting of neighboring regions which have a similar activity (Grova et al., 2006). Given an order of extension and a seed region, randomly chosen, neighboring regions of the seed are aggregated to the extended region, up to the order of extension. More formally: let us note *𝒮* the set of sources in the source space and *s*∈*𝒮* be a randomly chosen seed source. The first-order neighborhood of *s*, denoted *𝒱*^(1)^(*s*), is the set of sources that share an edge with *s* in the sub-sampled cortical mesh. To create a region of order *o*, we recursively compute


$$\begin{array}{l} {𝒱}^{\left(k\right)}=\left\{{𝒱}^{\left(k\right)}\left(s\right)\cup \left\{{𝒱}^{\left(k-1\right)}\left(s^{\prime }\right);s^{\prime }\in {𝒱}^{\left(k-1\right)}\left(s\right)\right\}\right\}, \end{array}$$

for *k*∈[[2, *o*]]. The number of extended regions is 2 and the extent order is randomly selected in the range 1–3.

Temporal patterns are generated using event related (ER) activity simulation, taking the form of a Gaussian function over time


(5) $$\begin{array}{l} f\left(t\right)=ae^{-\frac{1}{2}{\left(\frac{6\left(t-c\right)}{w}\right)}^{2}}, \end{array}$$

where *a* represents the amplitude, *c* the center and *w* = 6σ the width of the Gaussian. In a given extended region, all sources share the same temporal signal, with the amplitude decreasing according to a Gaussian function of the distance from the considered region to the seed region. In this work, the amplitude is randomly selected in the range 0.5 − 1.5 nA m, divided by the mean number of sources in the region (20.6), center and width are selected in ranges 49 − 51 ms and 125 − 375 ms respectively. Each example has a 1 s duration with a sampling frequency of 500 Hz, i.e 500 temporal samples: $\text{X}\in {\mathbb{R}}^{N_{s}\times 500}$.

A noiseless EEG signal $\tilde{\text{Y}}\in {\mathbb{R}}^{N_{e}\times T}$ is generated by projecting a source distribution **X** onto the electrode space through the forward model.

Noise is added to the EEG data at a given signal to noise ration (SNR). Given a white Gaussian noise $\epsilon \in {\mathbb{R}}^{{\mathbb{N}}_{�}\times T}\$,\$\epsilon \sim N(0,I)$, the SNR is set by computing


(6) $$\begin{array}{l} \text{Y}=\tilde{\text{Y}}+\frac{\epsilon }{||\epsilon |{|}_{F}}\frac{||\tilde{\text{Y}}|{|}_{F}}{\sqrt{SNR}}. \end{array}$$

The SNR is set to 5 dB.

##### 2.2.1.3 Data simulation using biophysical models (NMM)

The second type of EEG data simulation relies on Neural Mass Models (NMM), that are mathematical representation of the interaction between populations of neurons. They can be used for the study of the functional behavior of the brain without having to model the interactions at the level of single neurons (David and Friston, 2003).

The model used is a Jansen-Rit model (Jansen and Rit, 1995) which is based on the interaction between three subpopulations within a cortical column: the pyramidal cells, excitatory, and inhibitory interneurons. In this model, for default values of parameters, the model generates an alpha-like activity. When the gain A, i.e., average excitatory synaptic gain, is increased to about 3.5-3.6 for a region, spike-like activity appears in this region.

Following Sun et al. (2022), source distributions are simulated using The Virtual Brain (TVB) (Sanz Leon et al., 2013). To simulate a source distribution, one region was selected as active and its gain value A is set to 3.6. The other regions retained the model's default parameters. This process is repeated, treating each region in turn as an active region. Data was simulated at a 2,000 Hz frequency rate and subsampled at 500 Hz frequency rate.

Post processing is done (in Matlab) in order to extract portion of one second of signal containing a spike for the active region and no spike in other regions. The data is scaled to obtain a SNR of 15 dB between the source and background activities.

To generate a source distribution, a seed region is chosen. Neighboring regions are aggregated to the seed in a random direction [details in the article (Sun et al., 2022)], to a certain order of extension similar definition given in the previous section. This defines the spatial pattern. For the temporal pattern, a random spike is selected from those extracted from the raw data where the active region corresponds to the considered seed region. The waveform of the spike is assigned to the selected neighboring regions, with a decrease in magnitude as a function of the distance. Finally white Gaussian noise is added to the EEG data with a SNR of 5 dB, 10 dB, 15 dB and 20 dB.

To generate the dataset, 6 portions of 10 seconds each of source activity are generated for each region (for the active region A = 3.6 and A = 3.25 for all non active regions; mu = 0.087, sigma = 1). Two extended regions are used, with an extension order of 3.

**Figure 2:**
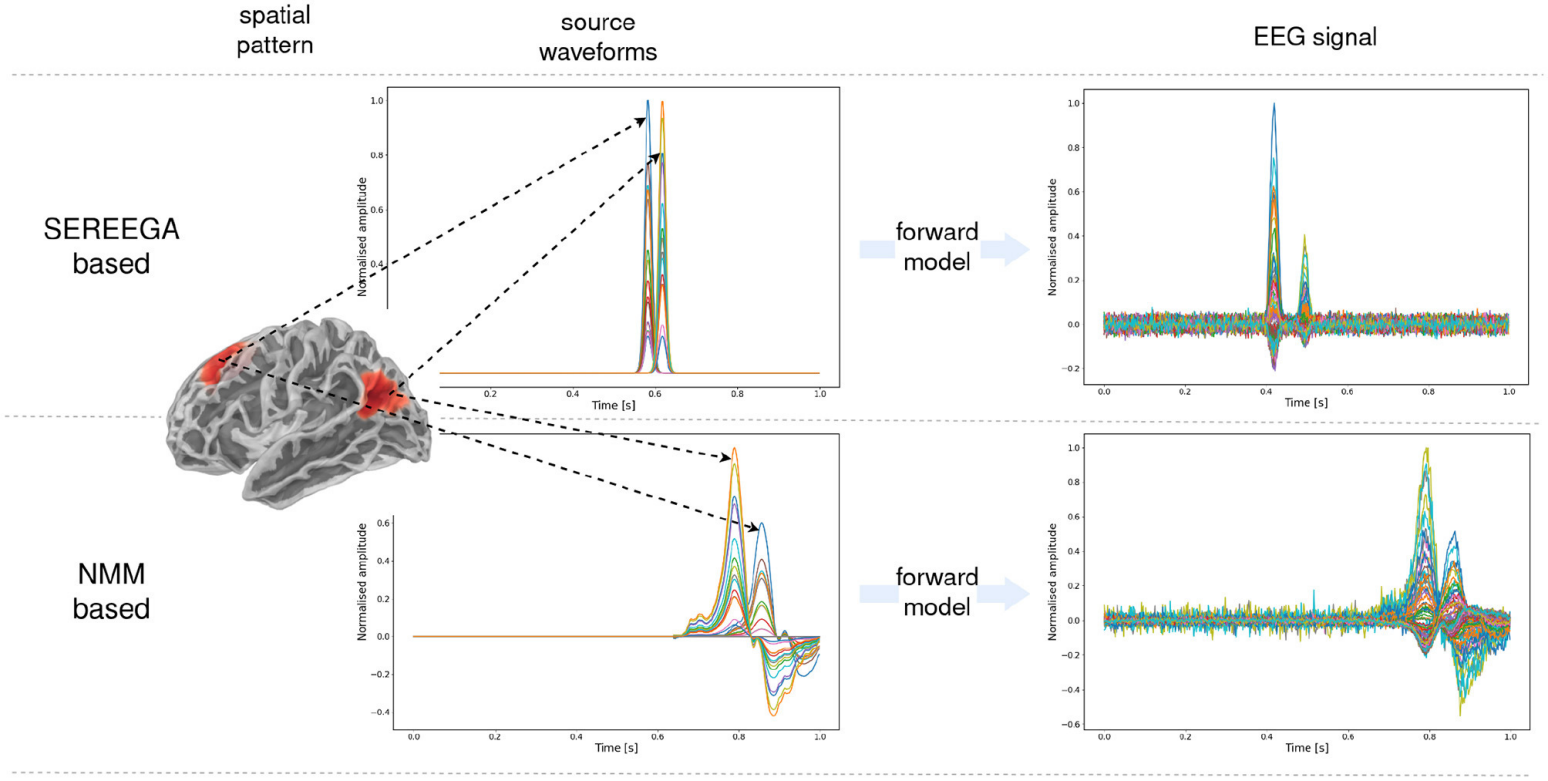
Simulated data with the two different types of simulation: SEREEGA based and NMM based. Visualization of the waveform of active sources and the final EEG data obtained through the forward model. For the NMM based simulation, the simulated source activity contains signals for active sources (spikes) and noise sources. The generated EEG corresponds to the projection of this “noisy” source activity. However the ground truth source data that is used for training corresponds to a “denoised” source activity where all non-active regions are set to zero.

Figure 2 illustrate the two types of simulations, the similarity in the spatial pattern and differences in waveform and noise simulation.

#### 2.2.2 Real data

**Figure 3:**
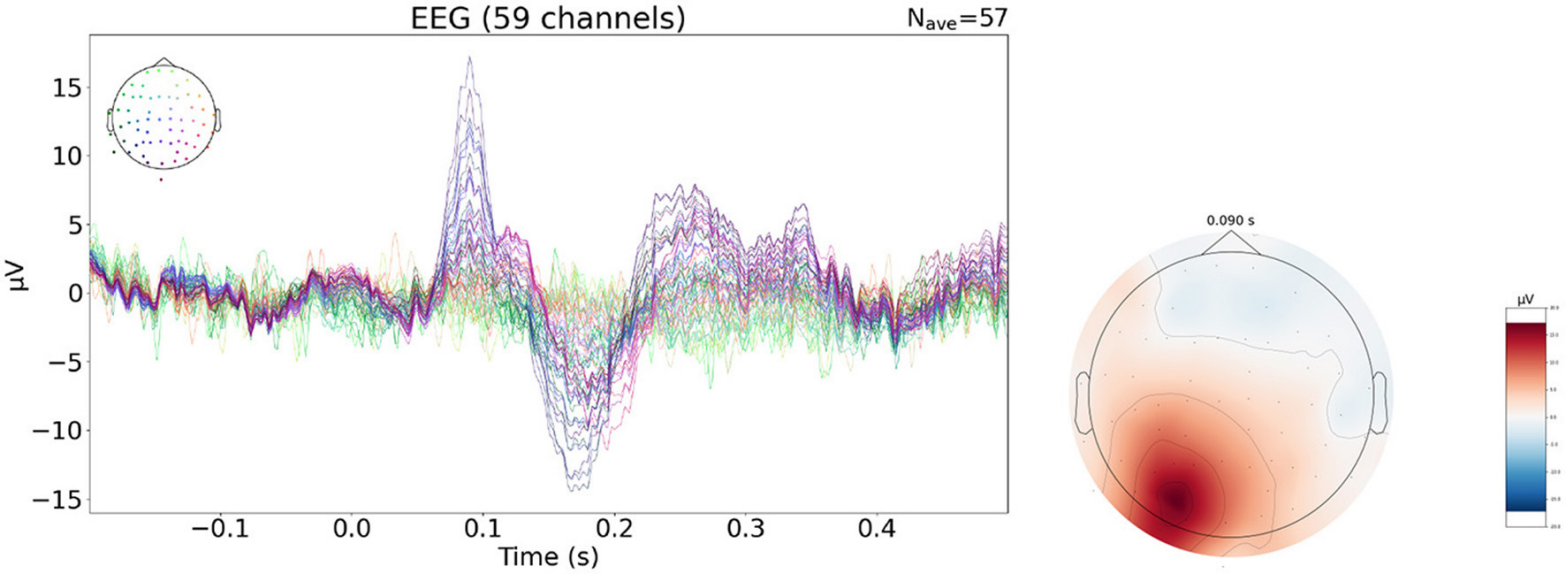
Evoked data of visual right evoked potential. Topographic map at the time of the P100 (90 ms).

To further complement this study, we evaluate performances of neural networks on a publicly available real dataset of evoked potential activity. The use of evoked potential data allows to remain close to the type of activity of the synthetic dataset used to train the networks. This real EEG data is available through mne-python (Gramfort et al., 2013) and is shown in Figure 3. In the experiment, different stimuli are presented to the subject in order to elicit different brain responses. We focus on the response to visual stimulation in the right visual field.

The raw EEG data contains 61 electrodes but two channels are dropped due to poor signal quality, resulting in a 59 electrode setup. Minimal pre-processing is done on the data: average referencing is applied and epochs with an amplitude superior to 150 mV are removed. The remaining epochs of the visual right condition (*N* = 57) is averaged, time locked to the stimulus event, to obtain the evoked data. The signal obtained is 421 samples long with a 600.6 Hz sampling frequency i.e around 700 ms of signal of which 200 ms are baseline data and 500 ms correspond to the response to the stimulus. The neural networks are trained using SEREEGA based simulations for which no connectivity matrix is needed. The head model was built from the MRI of the subject. The cortex mesh is subsampled to obtain a 1,176 = vertices mesh. Sources are oriented perpendicular to the surface. The solver used is a BEM with 3 surfaces: the brain, the skull and the skin, with respective conductivity values. The electrode montage is made of 59 channels from the EEG recording. The size of the leadfield matrix is 59 × 1,176. The training data has the same temporal characteristic as previously described. The spatial pattern is changed: the number of regions is selected between 1 and 3 instead of being fixed to 3, and the extension order is selected in the range 1–5.

#### 2.2.3 Neural network training

The networks were trained on 500 epochs using the ADAM optimiser with default parameters and a batch size of 8, using PyTorch and PyTorch Lightning (Paszke et al., 2019; Falcon and The PyTorch Lightning team, 2019). Early stopping was applied if the validation loss function did not decrease for 20 epochs. Gradient clipping was used for the LSTM's training, to avoid gradient vanishing or exploding. The number of parameters for each network, and for the two head models used are given in Table 1.

**Table 1:** Number of parameters of the 3 neural networks studied, for the simulated dataset (head model with 994 regions) and the real data experiment (head model with 1,176 sources).

**Network**	**Number of parameters**	
	*N*_*s*_ = **994**	*N*_*s*_ = **1176**
1D-CNN	5,177,344	6,025,216
LSTM	461,380	473,960
deepSIF	22,380,956	30,607,824

#### 2.2.4 Evaluation metrics

ESI focuses on the estimation of spatio-temporal source activity given EEG signals.

In an ideal reconstruction, the solution would accurately estimate amplitude vectors, for each time point, enabling the precise localization of active sources in the brain. More precisely, in the case of a source distribution with a single extended source/region, the goal is to properly estimate the source/region with maximum activity (seed source/region), the extension of the active area and the amplitude of sources (Hauk et al., 2022; Samuelsson et al., 2021).

In this work, we consider 5 complementary metrics to provide a comprehensive analysis of the performance of the neural networks evaluated: the localization error (LE), the area under the ROC curve (AUC), the time error, the normalized mean squared error (nMSE), and the peak signal to noise ratio (PSNR). The LE, AUC, time error, and nMSE are computed at the time of the maximum activity of the ground truth seed source/region while PSNR is computed over the entire temporal dimension to provide a more global information.

**Localization error (LE)**, measured in mm, assesses the ability of a method to estimate the position of active sources. It is defined by the Euclidean distance between the true maximum source, denoted as *s* and positioned at **r**_**s**_, and the estimated maximum source, denoted as ŝ and positioned at ${\text{r}}_{\hat{\text{s}}}$


(7) $$\begin{array}{l} LE\left({\text{X}}_{:,t_{0}},{\hat{\text{X}}}_{:,t_{0}}\right)=||{\text{r}}_{\text{s}}-{\text{r}}_{\hat{\text{s}}}|{|}_{2}. \end{array}$$

The **Area Under the ROC (receiver operating characteristic) curve (AUC)** provides information about a method's ability to accurately recover a source's extent. In the context of ESI, the AUC, expressed in %, assesses the method's capability to precisely determine the source's extension and not to create spurious activity. Usually the AUC is calculated to measure the accuracy of a classifier. It is the area under the ROC curve. The ROC curve is the curve of the true positive rate (TPR) as a function of the false positive rate (FPR), for different threshold values. To compute the AUC in our situation, the source distribution's absolute value is scaled between 0 and 1 and then thresholded, enabling a binary classification task of active and inactive source classes. Then the TPR is defined as $TPR=\frac{TP}{TP+TN}$ and the FPR as $FPR=\frac{FP}{TN+FP}$, where TP are the true positives, i.e the estimated and ground truth sources are considered active, TN the true negatives, i.e., the estimated and ground truth sources are considered as inactive, and FP the false positives, i.e., the estimated source is considered inactive while the ground truth source is active.

To evaluate the ability of a method to properly estimate the amplitude of a source distribution, we use the nMSE and the PSNR. **nMSE** is the mean square error between the estimated normalized source distribution and the ground truth. Normalization is performed by dividing the source distribution by its maximum absolute amplitude value. Normalizing the source distribution facilitates the comparison of metric values between different methods. It considers the estimated overall pattern instead of the precise amplitude values. nMSE at a time *t*_0_ is given by:


(8) $$\begin{array}{l} nMSE\left({\text{X}}_{{\text{:,t}}_{\text{0}}},\hat{{\text{X}}_{{\text{:,t}}_{\text{0}}}}\right)=\frac{1}{N_{s}T}\overset{N_{s}}{\underset{i=1}{\sum }}{\left(\frac{{\text{X}}_{{\text{i,t}}_{\text{0}}}}{\text{max}\left(\left|\text{X}\right|\right)}-\frac{\hat{{\text{X}}_{{\text{i,t}}_{\text{0}}}}}{\text{max}\left(\left|\hat{\text{X}}\right|\right)}\right)}^{2}. \end{array}$$

The second measure for assessing the estimation of the amplitude is the **PSNR**. It is calculated between the normalized source distributions over the entire duration of the source distribution. PSNR is computed as:


(9) $$\begin{array}{l} PSNR\left(\text{X},\hat{\text{X}}\right)=10\log_{10}\left(\frac{\underset{i,t}{\text{max}}{\left({\tilde{\text{X}}}_{i,t}\right)}^{2}}{MSE\left(\tilde{\text{X}},\tilde{\hat{\text{X}}}\right)}\right). \end{array}$$

where $\tilde{\text{X}}=\text{X}/$max(|**X**|) and $\tilde{\hat{\text{X}}}=\hat{\text{X}}/$max$\left(\left|\hat{\text{X}}\right|\right)$

Finally the **time error** offers information about the ability of a method to correctly estimate the source waveform (temporal activity). It is determined by calculating the absolute difference between the instant of maximum activity of the ground-truth activity and that of the estimated source's (i.e., the instant of maximum amplitude of the estimated seed source). The temporal error, measured in *ms*, is computed as $TE=|t_{\max}-{\hat{t}}_{\max}|$, where $t_{\text{max}}=\underset{t}{\text{argmax}}\sum_{\text{i=1}}^{{\text{N}}_{\text{s}}}{\text{|X|}}_{\text{i,t}}$.

Table 2 provides an overview of the 5 metrics considered in this study.

**Table 2:** Assessment metrics used in this work and the corresponding evaluated aspects of the estimated solutions.

**Metric**	**Aspect of the source distribution that is evaluated**
Localization Error (LE)	Localization
Normalized MSE (nMSE)	Amplitude
AUC	Extension
Time error (TE)	Temporal
PSNR (on multiple time instants)	Amplitude

The localization error, normalized MSE and AUC are calculated for a single time point while the PSNR is calculated over the entire time series.

## 3 Results

This section describes results for three different experimental settings: (1) multiple extended sources, (2) out of domain generalization, (3) application to real data. Results of the three considered deep learning-based methods are compared to two non-learning based methods: MNE and sLORETA.

### 3.1 Estimation of multiple extended sources—SEREEGA and NMM based simulations

This first experiment evaluates the performance of the algorithms in a standard supervised learning framework. Each simulated dataset contains 10,000 samples and is separated into two datasets: training set (80%), validation set (20%). This training-validation splitting strategy is the same for all experiments. Methods are evaluated for both simulated datasets: SEREEGA and NMM. Quantitative results are given in Table 3. Whatever the evaluation metric used, deep learning-based methods outperform non-learning based methods. Statistical analysis was performed to determine whether the difference in performance between the learning and non learning methods was significant. ANOVA was performed on the different methods, separately for each metric, showing a significant difference between the methods for each metric. To further investigate which of the methods are significantly different, we performed a *post hoc* Tukey test, which provides pairwise statistical significance between methods. All deep learning methods appeared to be statistically different from sLORETA (the best non-learning based method) for all metrics except time error. For the networks trained and evaluated on the SEREEGA dataset, the LSTM, and deepSIF models are not statistically different from sLORETA (*p*-value = 0.623, *p*-value = 1) on time error. However, 1D-CNN is significantly better than sLORETA (*p*-value ≤ 0.05). For the models trained and evaluated on the NMM based dataset, all learning based methods have a worse performance than sLORETA in terms of time error, but the difference is not significant for the LSTM model (*p*-value = 0.051). In this experiment, the deep learning-based method that provides the best results is deepSIF. However, for the localization error the results are not statistically different from those obtained with the 1D-CNN model (for both datasets, *p*-value = 0.14 and *p-*value = 0.06). These results have to be interpreted in light of the number of parameters in each network. It should be noted that deepSIF is a network with tens of millions of parameters, whereas the LSTM architecture is much lighter, with less than 5 × 10^5^ parameters. The 1D-CNN model represents a compromise between the other two models. In order to study a possible bias linked to the data used, the second experiment looks at the networks' ability to generalize.

**Table 3:** Evaluation results for the 3 learning based methods and 2 non-learning based methods.

**Methods**		**1DCNN**	**LSTM**	**deepSIF**	**MNE**	**sLORETA**
**Data**	**Metrics**					
SEREEGA	LE [mm]↓	2.99	5.17	**2.64**	14.51	9.17
	AUC (%) ↑	81.39	82.19	**87.65**	73.08	75.59
	nMSE ↓	0.002	0.002	**0.001**	0.017	0.040
	PSNR ↑	43.41	40.80	**43.43**	30.40	26.93
	time error [ms] ↓	**1.35**	3.57	4.58	9.55	4.72
NMM	LE [mm]↓	11.30	**10.07**	10.80	15.65	12.12
	AUC (%) ↑	80.36	87.90	**88.72**	75.65	77.59
	nMSE ↓	0.012	**0.008**	**0.008**	0.020	0.042
	PSNR ↑	31.54	33.18	**33.42**	29.23	25.83
	time error [ms] ↓	217.71	196.01	**183.32**	184.57	167.25

Simulation type are similar for training and testing: neural networks trained on SEREEGA based data are tested on a SEREEGA based test dataset, and the same is done for NMM based data. Metrics are computed on 2,000 samples. Bold font indicates best results.

### 3.2 Out of domain generalization

The second experiment focuses on the out-of-domain generalization of the networks to other types of simulations. We evaluate the networks trained on SEREEGA (respectively NMM) based simulated data and evaluate the results on NMM (respectively SEREEGA) based simulated data. EEG signals from these datasets are generated from different processes, leading then to a suitable experimental setup to assess over-fitting behavior. Quantitative results are given in Table 4. In line with previous experiment, methods based on learning techniques generally provide better results than non-learning methods. However, the performance improvement is less pronounced and the sLORETA method obtains the best scores for some metrics. More specifically, the same statistical analysis as in Section 3.1 was performed on these results, for each metric separately. The localization error performance for the networks trained on SEREEGA and tested on the NMM dataset is significantly better for 1D-CNN, compared to all other methods. The time error is worse for 1D-CNN and LSTM than for sLORETA, but this difference is not statistically significant (*p*-value = 0.24, *p*-value = 0.38). For deepSIF, the performance is significantly better. For the networks trained on NMM and tested on the SEREEGA dataset, sLORETA performs significantly better for the localization error. For the time error, sLORETA also performs significantly better than all other methods except for CNN, for which the difference is not significant (*p*-value = 0.98). For all other metrics and pairs of methods, the difference was found to be significant (*p*-value ≪ 0.05).

**Table 4:** Results for out of domain generalization: NNs are trained on one type of simulation and tested on the other type of simulation.

**Methods**		**1DCNN**	**LSTM**	**deepSIF**	**MNE**	**sLORETA**
**Data**	**Metrics**					
SEREEGANMM	LE [mm]↓	**7.38**	9.39	11.86	15.65	12.12
	AUC (%) ↑	76.81	74.03	74.43	75.65	**77.59**
	nMSE ↓	**0.007**	**0.007**	0.009	0.020	0.042
	PSNR ↑	32.17	32.46	**32.58**	29.23	25.83
	time error [ms] ↓	178.95	156.99	**129.47**	184.57	167.25
NMMSEREEGA	LE [mm]↓	11.35	12.41	11.36	14.51	**9.17**
	AUC (%) ↑	77.68	**85.37**	81.52	73.08	75.59
	nMSE ↓	0.015	0.011	**0.006**	0.017	0.040
	PSNR ↑	35.03	36.80	**40.24**	30.40	26.93
	time error [ms] ↓	5.19	8.42	15.89	9.55	**4.72**

In the first experiment, SEREEGA is used to generate training data and NMM is used for evaluation. For the second experiment, the roles of the data simulators are reversed. Bold font indicates best results.

**Figure 4:**
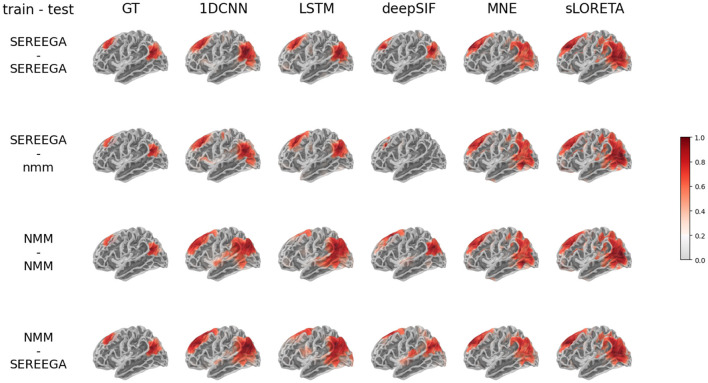
Results on simulated data for the two different data simulation type : based on physical model (SEREEGA) and biophysical model (NMM). **First row**: neural network trained on the SEREEGA dataset and evaluated on SEREEGA simulation. **Second row**: neural network trained on the SEREEGA dataset and evaluated on NMM simulation. **Third row**: NN trained on the NMM based dataset and evaluated on NMM baesd simulation. **Fourth row**: NN trained on the NMM based dataset and evaluated on NMM simulation. The source distribution plotted corresponds to the mean of the source distribution at the instant of maximum activity of each region, in absolute value and normalized between 0 and 1.

This quantitative assessment is supplemented by a visual analysis provided by the Figure 4. Given a simulated ground truth activity (left column), the predictions of each method are displayed as a function of the training data and the test data. These visual results illustrate the variety of results, in particular predictions that are more spread out than the ground truth or the absence of estimated activity (such as deepSIF when trained on SEREEGA data and evaluated on NMM data).

### 3.3 Real data

**Figure 5:**
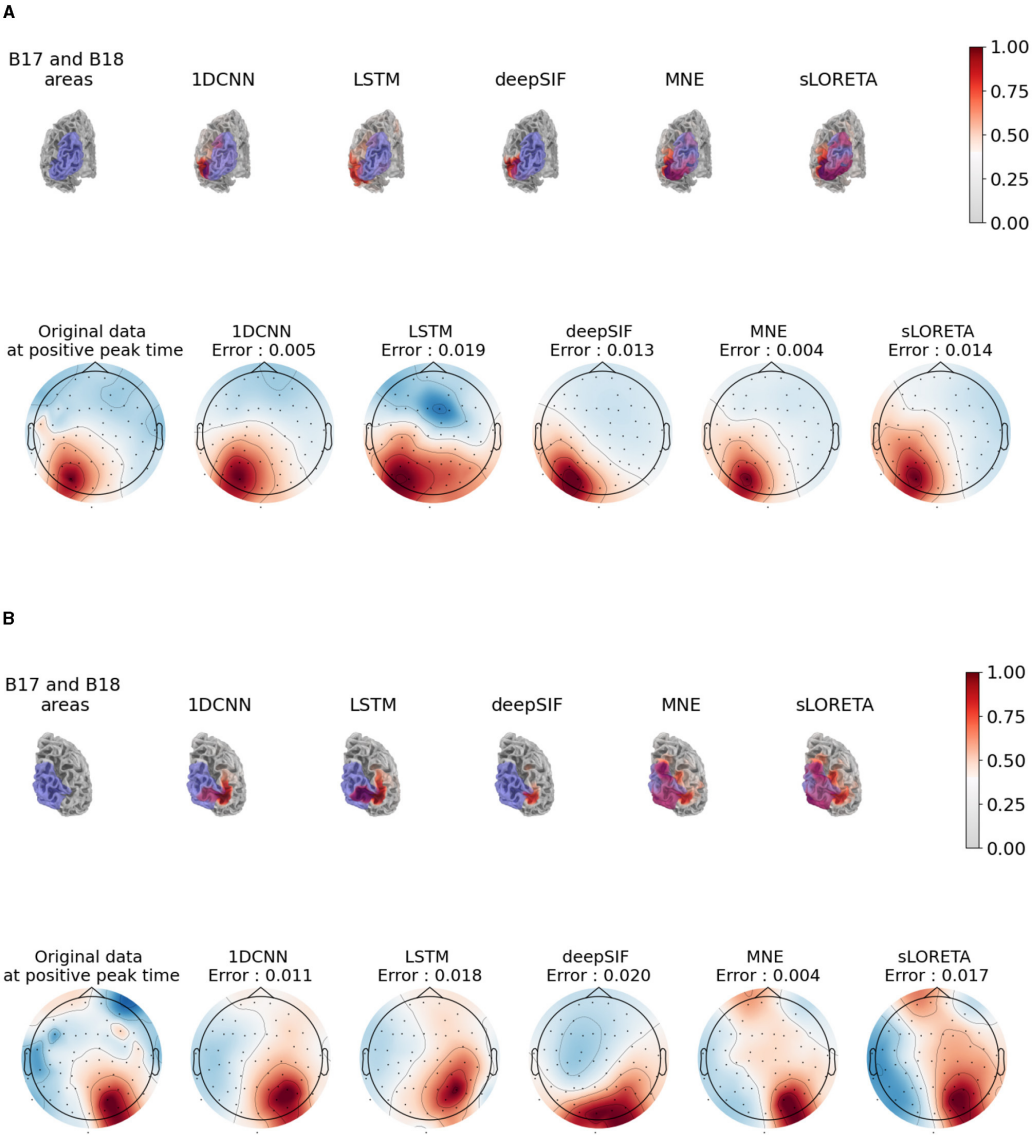
Results of source estimation for the non-learning and learning based methods on the real data from the *sample* dataset (Gramfort et al., 2013). For each subfigure, the top row corresponds to the visualization of the absolute value of source activity at the instant of the P100, on the subject mesh. The purple area corresponds to the B17 and B18 Broadman visual area (morphed from a template atlas). A threshold of 20% is applied on this visualization. The bottom row shows the re-projection of the estimated activity onto the scalp through the leadfield matrix vs the ground truth EEG data, also at time of the P100. **(A)** Results for the evoked potential corresponding to the “right visual stimuli” condition. **(B)** Results for the evoked potential corresponding to the “left visual stimuli” condition.

The last experiment consists of studying the results of source estimation on real EEG data, using a network trained on simulated data. We focus on the evoked EEG data in response to a stimulus in the right (or left), visual field. The waveform contains a P100 component that is expected to originate from the left (or right) visual area of the brain (Di Russo et al., 2002), in the B18/V3 area of the brain. Since no ground truth is available for the real data, qualitative evaluation is done through the visualization of estimated activity. The estimated source activity for the five methods is given at the top of Figure 5A for the right visual stimuli and Figure 5B for the left visual stimuli.

We also projected the estimated source back onto the electrodes through the leadfield matrix and calculated the reprojection error, i.e., the nMSE between the true EEG data and the estimated EEG data. Such a visualization provides a way to assess the quality of the estimated activity by analyzing the discrepancy in the EEG data space. The topographic plots are shown at the bottom of Figures 5A, B, compared to the acquired EEG data.

We can see on the source estimate plots that the MNE and sLORETA solutions are quite spread out, but have a high absolute value in the visual area, especially in area B18, which is consistent with theoretical findings on the sources of the P100. The results of the different neural networks are similar, with a high level of activity close to the left visual area. However, this activity is not in the area, but shifted to the left (respectively right). On the topographic maps of the reprojection of the estimated source activity, we can see that the 1D-CNN and deepSIF methods are closer to the acquired EEG data than LSTM.

## 4 Discussion

ESI is a valuable technique for non-invasively analyzing brain activity and dysfunction. However, due to the inherent ambiguity in the ESI problem, most ESI methods incorporate regularization terms into their optimization objectives, which impose constraints on the solution space to enhance its reliability and interpretability. While sLORETA, a widely used method based on Euclidean norms, offers computational efficiency, its source estimates often exhibit excessive spatial spread, potentially obscuring the precise underlying neural activity.

Deep learning approaches can provide an alternative to solve these ill-posed source imaging problems by incorporating biophysical models into ESI solutions through the creation of dedicated simulation data. This study explored the potential of data-driven learning methods for ESI, aiming to overcome the limitations of conventional methods that rely on explicit priors on source localization. To achieve this, we employed a large dataset of sensor-source mapping examples, carefully designed to capture the diverse patterns of brain activity distributions. The distribution of this training dataset was tailored to closely resemble the actual signals that the learning network would encounter during its intended use.

In this work, we considered 3 learning methods with diverse architectures in order to provide an overview of the capabilities of these methods for ESI compared to state-of-the-art methods (MNE and sLORETA). The performance of these methods significantly hinges upon the training dataset. To evaluate their performance, we employed a set of five complementary metrics that comprehensively assess various aspects of source reconstruction accuracy. Additionally, we utilized two synthetic datasets, SEREEGA, and NMM, generated using state-of-the-art methods, to evaluate their performance and generalization capability. One of the limitations of the use of synthetic data to train neural networks is that the solution is highly dependent on the underlying hypothesis made on the data used to train the networks, and thus careful interpretation of the results should be made. The experiments underscored the dependency of these methods on training data, demonstrating reduced performance when the training data deviates significantly from the evaluation data. Future work should focus on reducing this drop in performance, either by finding a more suitable architecture or by creating a more diverse dataset.

This in-depth investigation was further complemented by a study conducted on real-world data, further demonstrating the promising capabilities of learning-based approaches for ESI. This study also highlights the crucial need to incorporate confidence measures alongside deep learning predictions to ensure the reliability of the obtained results. This could be done for example using methods such as Monte Carlo dropout (Gal and Ghahramani, 2016) to get information about the uncertainty of the model on the source estimation. More work should also be done in order to add other real data experiments.

Making quantitative comparisons between different studies in ESI research is challenging due to the inherent differences in the simulated data and methodologies used. A significant source of variation arises from the MRI data employed to create head models, which differ between studies in terms of the number of sources and electrodes. Additionally, the models used to generate brain electrical activity vary considerably. For instance, some studies, including (Hecker et al., 2022, 2021) and the present work, utilize physical models of waveforms, while others adopt biophysical models such as neural mass models (NMMs) (Sun et al., 2022). Furthermore, some studies extract temporal patterns for sources using real EEG waveforms (Wei et al., 2021), whereas others, such as Jiao et al. (2024, 2022), operate at single time instants, thus avoiding waveform simulation entirely.

In this study, we addressed this variability by comparing two distinct types of simulated data, each generated using different models, to evaluate performance across diverse datasets and assess the generalization abilities of the models. Another challenge in making comparisons between studies lies in the inconsistency of metrics used. Even when similar metrics are employed, they are often calculated differently. For example, localization error, a commonly used metric in source imaging literature, is difficult to compare directly due to variations in its calculation across studies.

A common characteristic across different studies is their focus on reconstructing single or multiple extended sources. The accurate estimation of extended and multiple sources remains a significant challenge in source distribution estimation. Consistent with previous findings (Sun et al., 2022; Huang et al., 2022; Jiao et al., 2022, 2024; Hecker et al., 2021, 2022; Wei et al., 2021), we observed that neural network-based methods outperform traditional non-learning methods, such as sLORETA and MNE, in recovering the spatial extent of sources. The latter tend to overestimate source extension, a well-documented consequence of the L2 norm regularization term.

Some studies (Sun et al., 2022; Huang et al., 2022) also highlight the inverse problem, where methods that promote sparisity, such as FOCUSS (Gorodnitsky et al., 1995)—which are not examined here—tend to underestimate source extension. These observations have been validated across a range of real datasets. For instance, models in Sun et al. (2022) and Jiao et al. (2022, 2024) demonstrated superior performance in estimating source extension using epilepsy data, while models in Hecker et al. (2021), Sun et al. (2022), and Wei et al. (2021) consistently showed robust results across various types of evoked data.

The findings from this study, particularly regarding generalization, align with these earlier results. Neural networks trained on the SEREEGA dataset, when tested on NMM-based data, exhibited superior performance in estimating source extension compared to sLORETA and MNE. Additionally, they provided a more focal representation of the source on real data. This suggests that *post hoc* thresholding, commonly applied to source estimates (Maksymenko et al., 2017), is less error-prone or potentially unnecessary for learning-based methods in comparison to traditional non-learning-based techniques.

In this study, we focused on a multiple-source dataset, which presents a significant challenge for source estimation. Previous studies that have utilized both single and multiple extended sources have shown that as the number of sources increases from one to multiple, the performance of all methods declines. However, this decline is more pronounced in non-learning-based methods (Hecker et al., 2022).

These findings are highly encouraging and highlight the potential of supervised learning methods in addressing the challenges posed by ESI, particularly in accurately recovering the source extent for both single and multiple active sources. However, this study also underscores the limitations of the approach, specifically the reliance on simulated data. The type of simulation used significantly influences the results and the generalization ability of the models, which may pose challenges when translating these results to real-world applications.

In conclusion, our analysis has provided a comprehensive assessment of data-driven deep learning methods for ESI. Deep learning methods can provide a powerful framework for ESI and improve its adoption for spatiotemporal dynamic imaging of the human brain, facilitating the clinical diagnosis and treatment of a variety of neurological and mental diseases. Future key research directions for enhancing the reliability and interpretability of deep learning-based ESI are confidence estimation and sensitivity analysis at the individual level. Regarding the method, future work could focus on variational approaches linked with deep learning approaches such as done in Jiao et al. (2024) for ESI, or in Fablet et al. (2021) on another type of dataset. In these approaches more explainability and flexibility is given to the model by learning the prior with a neural network while keeping an observation term which takes into account the forward model.

## Data Availability

The datasets presented in this study can be found in online repositories. The names of the repository/repositories and accession number(s) can be found below: https://github.com/SarahReynaud/stESI_pub.
